# Magnetic Force Probe Characterizations of Nanoscaled Ferromagnetic Domains: Finite-Element Magnetostatic Simulations

**DOI:** 10.3390/nano12132212

**Published:** 2022-06-28

**Authors:** Xiao-Xia Zheng, Wei-Feng Sun

**Affiliations:** 1 College of Computer Science and Technology, Heilongjiang Institute of Technology, Harbin 150050, China; xiaoxia_hit@126.com; 2 School of Electrical and Electronic Engineering, Nanyang Technological University, Singapore 639798, Singapore

**Keywords:** magnetic force microscopy, ferromagnetic nanomaterial, magnetic domain, finite-element simulation

## Abstract

Microscopic characterization of magnetic nanomaterials by magnetic probe interacting with ferromagnetic nano-domains is proposed according to finite-element magnetostatic field simulations. Magnetic forces detected by microscopic probe are systematically investigated on magnetic moment orientation, magnetization intensity and geometry of ferromagnetic nano-domains, and especially on permanent magnetic coating thickness and tilting angle of probe, to provide a theoretical basis for developing magnetic force microscopy. Magnetic force direction is primarily determined by magnetic moment orientation of nanosample, and the tip curvature dominates magnetic force intensity that is meanwhile positively correlated with nanosample magnetization and probe magnetic coating thickness. Nanosample should reach a critical thickness determined by its transverse diameter to be capable of accurately detecting the magnetic properties of ferromagnetic nanomaterials. Magnetic force signal relies on probe inclination when the sample magnetic moment is along probe tilting direction, which, however, is not disturbed by probe inclination when sample magnetic moment is perpendicular to probe tilting plane. Within the geometry of satisfying a critical size requirement, the magnetic force can successfully image the ferromagnetic nano-domains by characterizing their sizes and magnetic moment orientations. The present study is expected to provide effective analyzing schemes and theoretical evidences for magnetic force microscopy of characterizing magnetic structures in ferromagnetic nanomaterials.

## 1. Introduction

Magnetic nanoparticles (MNPs) have unique physical properties such as superparamagnetism, macroscopic quantum tunneling of magnetization, size-dependent properties and exchange bias, which are promised in advanced applications of high-density data storage, spintronic and magneto-optic devices, biomedical engineering and energy harvesting [1,2]. Therefore, it is crucial to study how to accurately characterize magnetic performances of ferromagnetic nanomaterials under the limitations by the size and shape of MNPs. With a high resolution of <10 nm, magnetic force microscopy (MFM) exploits a permanent probe to sensitively detect the stray fields of magnetic nanomaterials, which is prospective for the future imaging characterization of magnetic nanostructures [3]. Magnetic forces of ferromagnetic nanomaterials in external magnetic fields depend on magnetic field distribution, geometry and intrinsic magnetic characteristics of samples. The process of characterizing MNPs benefits from the knowledge of magnetic properties depending on the geometry and crystallography of nanoparticle itself, which is a challenging endeavor due to the highly dependent spintronic structures on atomic structures that will be inevitably distorted on the tested sample surfaces [4]. Hence, it is of great technological interest to provide a geometrical interpretation of the appearance and enhancement of magnetism in MNPs [5].

The origin of magnetism lies in the orbital and spin motions of coupling electrons. According to how materials respond to magnetic fields, magnetic materials are classified into five groups: diamagnetism, paramagnetism, ferromagnetism, ferrimagnetism and antiferromagnetism. The first two groups exhibit no collective magnetic interactions without magnetic order, while the last three groups represent a long-range magnetic order below Curie temperature. Ferromagnetic and ferrimagnetic nanomaterials are highly focused on the diversity of their magnetic structures (magnetic domains) in nanoscale which render their unique magnetic features far from general micro-scale magnetic domains [6]. Magnetic microstructure of ferromagnetic materials is composed of many magnetic domains which will spontaneously magnetize to saturation in a definite orientation if their size decreases enough to form a whole magnetic domain called single-domain MNPs. Single-domain ferromagnetic nanoparticles have permanent magnetic moments of producing a highly heterogeneous stray field which greatly depends on the size and magnetization of these nanoparticles. Therefore, magnetic probe detection relies on probe tip sharpness and detection spacing, which restrains the precision of characterizing single-domain ferromagnetic nanoparticles. Accordingly, it is difficult to analyze the magnetic force bearing on MFM probe by analytical calculations, while numerical calculation is the best solution for studying magnetic field and magnetization in magnetic force probe characterizations. In order to analyze the microscopic magnetic behavior of MNPs by MFM imaging, it is essential to improve MFM model for characterizing single-domain ferromagnetic nanoparticles.

At present, the available theoretical models for describing magnetic interactions between detection probe and MNPs include point probe model, extended charge model and micromagnetism model. Point probe model simplifies probe tip as a point magnetic dipole to approximately calculate probe magnetic force, which ignores the geometry and idealizes the magnetization states of probe tip, leading to a reliable result agreeing well with experiments only under the condition that the decaying magnetic field leakages of probe and sample are comparable [7,8,9,10,11]. In comparison, the probe tip of extended charge model is regarded as a pyramid with each surface giving a magnetization oriented towards pyramid vertex, while the magnetization on probe tip is supposed to be uniformly distributed, thus leading to a distinctly higher evaluation of magnetic moment than point probe model [12,13]. Micromagnetism model based on micromagnetic theory is a hot topic of MFM technology. Compared with pyramid probe tip, the micromagnetism model has been successfully applied to simulate the MFM with a carbon nanotube probe coated by Co ferromagnetic film (CNT-MFM), which demonstrates that CNT-MFM can achieve a sufficiently high resolution to measure perpendicular magnetic recording disks [14]. Furthermore, Mansuripur modeled the magnetic coated tip as a tetrahedron by an ensemble of cube elements, and Oti proposed a model of 2D triangle structure, while Tomlinson performed micromagnetic simulations with a 3D pyramid model, all of which were successful micromagnetism model paradigms by solving Landau–Lifshitz–Gilbert equations of micromagnetic theory [15,16,17].

The magnetic images measured by MFM are mainly derived from the second-order gradient of magnetic forces which is the convolution of stray magnetic fields on ferromagnetic sample surface and probe magnetic moment, which cannot be utilized to elucidate microscopic magnetic structures and determine magnetic moment orientations of magnetic domains. With the deepening study of microscopic magnetic phenomena, MFM is not sufficient for qualitatively observing magnetic structures on magnetic surfaces, while magnetic imaging technology is expected to alternate from qualitative to quantitative characterizations. Microscopic magnetic structures on surfaces of ferromagnetic materials, such as the size and orientation distribution of surface magnetic domains, can be revealed by analyzing magnetic force images.

## 2. Simulation Methodology

### 2.1. Analysis Method of MFM

Magnetic interaction in MFM is a long-range magnetic dipole action, and the atomic force probe coated with a permanent magnetic film can detect a stray magnetic field by scanning with a constant speed above the surface of a magnetic sample. As a result, the magnetic domain structure that produces a stray magnetic field can be characterized by detecting the distribution of magnetic forces or magnetic gradients. Magnetic force of the permanent magnetic coat on probe surface is calculated by integrating Maxwell stress tensor of stray magnetic field ***H***_sample_ on sample surface. The integral is performed on volume *V* of the probe magnetic coat with a magnetic moment ***M***_tip_ to obtain magnetic force [18]:

(1) $$F=\mu_{0}\int \nabla (M_{\mathrm{tip}}\cdot H_{\mathrm{sample}})dV_{\;\mathrm{tip}}$$

**Figure 1:**
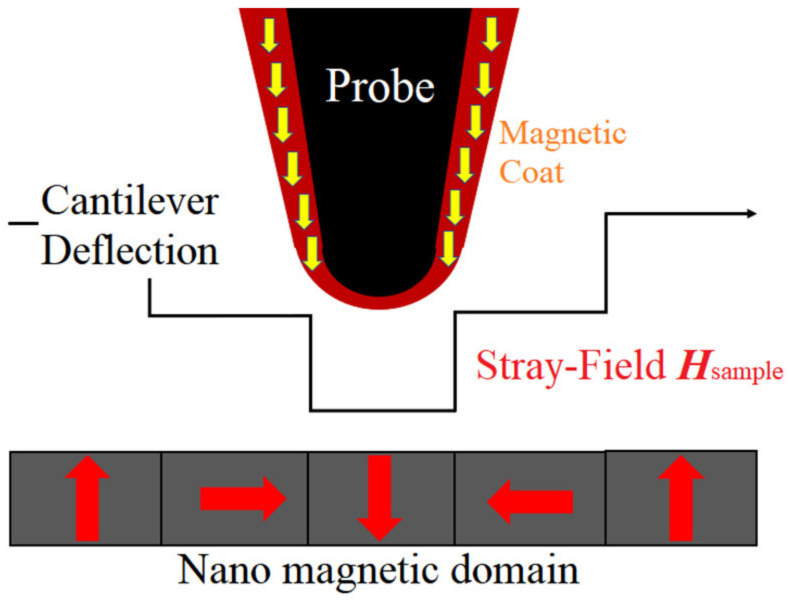
Schematics of magnetic force probe microscope.

Generally, magnetic tips are perpendicularly magnetized, and MFM is only sensitive to vertical component *H*_z_ of ***H***_sample_ and its derivative d*H*_z_/d*z*. As shown in Figure 1, probe cantilever deflection is mainly caused by the vertical component of *F* [19,20]:

(2) $$F_{z}=\mu_{0}\int (dH_{z}/dz)dV$$

To reduce interference from undesired stray fields to detection signals and improve measurement accuracy, the magnetic force variation Δ*F*(*z*) is used as a function of characterizing the magnetic domain structure as Δ*F*(*z*) *= F*_m_ − *F*_pre_ where *F*_m_ and *F*_pre_ denote the detected magnetic force at distances of *z* and 10 nm, respectively.

MFM numerical calculations require the coupling between magnetic field and mechanical strain to simultaneously calculate macroscopic deformation of probe cantilever in nanoscale and the turn-back of cantilever deflection to probe magnetic force [21]. For a preliminary evaluation, by coupling magnetostatics and structural mechanics, the interaction between the deflection of a steel cantilever and the probe magnetic force has been predicted to cause at most 10^−^^2^ nm error in the detection distances, which means that cantilever deflection could be ignored in the present nanoscaled MFM simulations. Magnetostatic field finite-element simulations in the dismissal of mechanical deformation are performed to investigate the magnetic forces depending on the magnetic moment orientation and thickness of nanosamples, and on the geometry and magnetic coat thickness of MFM probe.

### 2.2. Modeling Schemes

**Figure 2:**
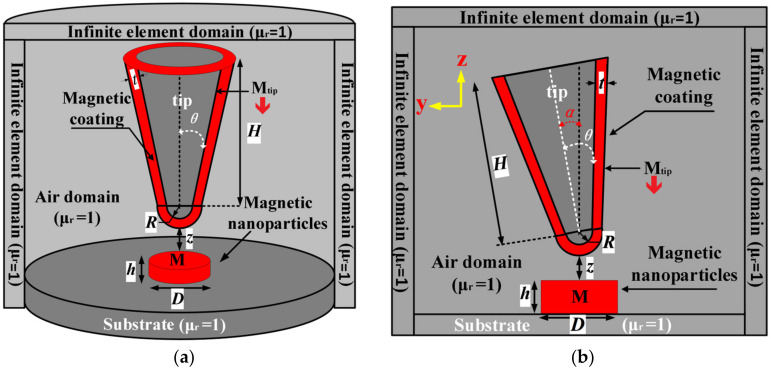
Schematic 3D model of magnetic force probe system: (**a**) vertical probe; and (**b**) tilt probe. *H*—truncated cone height; *R*—tip curvature radius; *D*—sample diameter; *z*—vertical distance from probe tip to sample surface; *h*—sample thickness; *θ*—probe cone half-angle; *α*—probe tilting angle; *t*—permanent magnetic coat thickness on probe surface.

Three-dimensional (3D) magnetostatic field simulations on magnetic force microscopy of characterizing ferromagnetic nanomaterials are performed as implemented by AC/DC module of COMSOL Multiphysics 5.6 package. As shown in Figure 2, MFM detecting system is modeled with an AFM probe and a single-domain ferromagnetic nanoparticle (magnetic nano-domain), and the probe surface is covered with a permanent magnetic coat [22]. The detecting probe consists of a truncating cone with a semi-angle of *θ*, a hemisphere tip with radius *R*, and a magnetic coat (CoCr) with a thickness *t* [23,24]. Probe tip is located vertically above the magnetic nano-domain. The magnetization intensity of probe magnetic coat is specified as 749 kA/m with the magnetic moment oriented along negative z-axis. Nanosample is set as a permanent magnetic nanoparticle (magnetic nano-domain) with a magnetization of 557 kA/m [25]. Environmental region around probe and nanosample is set as an air domain with a relative permeability of 1.

Periphery region and boundary are specified as infinite element domain and magnetic insulation, respectively. By free-tetrahedral method, the finite-element meshing near-probe tip is refined to a maximum size of 0.5 nm, in which the meshing growth rate and curvature factor are set to 1.1 and 0.2, respectively. Domain regions away from probe tip are meshed with a maximum element size of 5 nm. Finally, the magnetic force of probe is calculated by Maxwell stress tensor integral on probe surface according to Equation (2).

## 3. Results and Discussion

### 3.1. MFM Simulation Analyses of Single-Domain Ferromagnetic Nanoparticle

**Figure 3:**
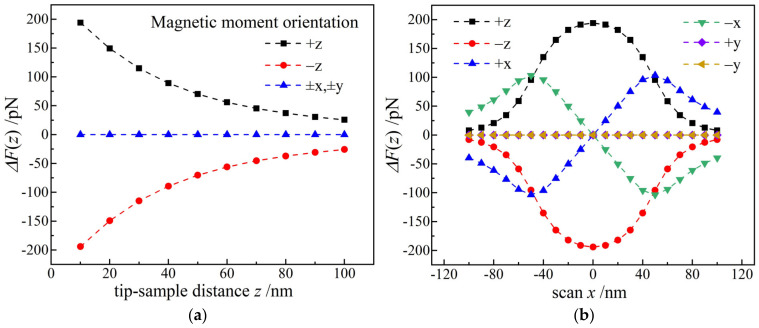
Probe magnetic force varying with (**a**) vertical and (**b**) horizontal scanning distance *x* for ferromagnetic single-domains (*D* = 100 nm, *h* = 100 nm) with different magnetic moment orientations.

Since magnetic moment of probe is critical for MFM measurements, it is required that magnetic moments distributed on probe magnetic coat should be strictly uniform to dismiss the stray irregularity of magnetic field on probe surface that may significantly reduce MFM resolution. The magnetic force detected by MFM is the vertical component of magnetostatic force between probe tip and sample, so MFM probe tip should be magnetized in the vertical direction (negative z-axis) to at most represent magnetostatic field [26]. Magnetic force on probe tip caused by ferromagnetic stray field is simulated by changing the magnetic moment orientation of single-domain nanoparticles (±z, ±x, ±y axes), as shown in Figure 3. The probed magnetic force signal decreases as the distance between probe tip and sample increases, and thoroughly relies on the magnetic moment orientation of sample. When the magnetic moments of probe and sample are parallel (negative z-axis), the magnetic force is attractive (red curve in Figure 3a), while for the single-domain sample with a magnetic moment vector perpendicular to probe magnetic moment (±x, ±y axes), the magnetic force is zero. In particular, magnetic force depends the direction of probe movement (scanning), as shown in Figure 3b. For the nano-domain sample with a magnetic moment oriented along ±z directions, the magnetic force will approach maximum when probe resides directly above sample (horizontal scanning distance *x* = 0). When the sample magnetic moment is in ±x directions, the magnetic force is a sinusoidal function of horizontal scanning distance *x* and will attain minimum at *x* = 0. Accordingly, the probed signal of magnetic force variations with the distance and direction of scanning probe can be exploited to characterize magnetic moment (intensity and orientation) of a magnetic nano-domain.

**Figure 4:**
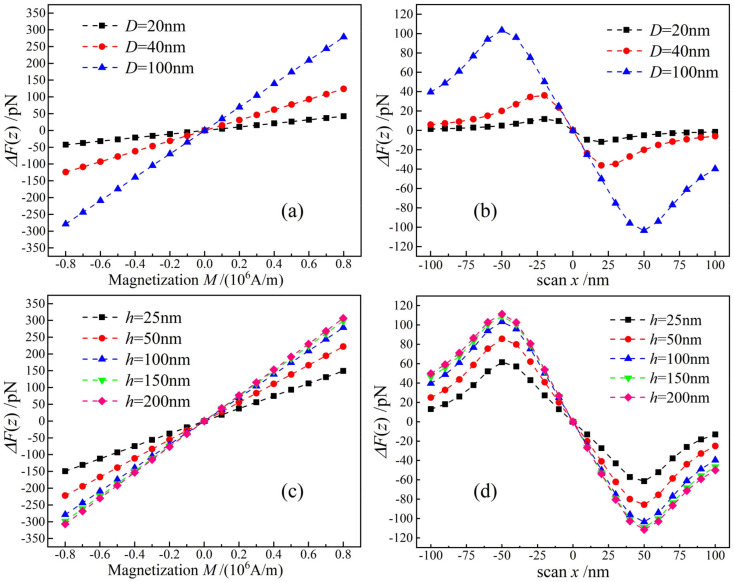
Magnetic forces versus magnetization of ferromagnetic single-domains with different sizes and magnetic moment orientations. (**a**) *h* = 100 nm, magnetic moment orientation +z; (**b**) *h* = 100 nm, magnetic moment orientation −x; (**c**) *D* = 100 nm, magnetic moment orientation +z; and (**d**) *D* = 100 nm, magnetic moment orientation −x.

Figure 4a,b show the curves of magnetic force signal changing with sample magnetization intensity and probe horizontal scanning distance. If the magnetic coercivity of probe is significantly lower compared with the magnetism of ferromagnetic sample, the probe magnetic moment will be influenced by the magnetic field from sample, while if the condition is reversed, the probe will dominate the ferromagnetic states of sample so that the detected magnetic signal of MFM cannot correctly represent magnetic characteristics of ferromagnetic nanomaterials [25]. For our models, we use numerical calculation method (finite-element difference method) to accurately solve stray magnetostatic fields without any approximations on probe geometry and magnetization which is more reliable than any analytical models [27]. It is indicated that the magnetic probe and nanosample of our MFM model are consistent to present a magnetic force that is accurately linear in dependence on magnetization intensity of sample, as shown in Figure 4a. By measuring magnetic force with horizontal scanning, the size of single-domain nanoparticle can be accurately quantified, as shown in Figure 4b, indicating that the distance between two magnetic force peaks identifies transverse diameter of magnetic nano-domain. Furthermore, transverse dimension (*D*) and thickness (*h*) of single-domain nanoparticles are also regulating magnetic force, as especially illustrated in Figure 4c,d that magnetic force is gradually saturated with increasing *h*. For the specific probe tip of *R* = 100 nm and the sample magnetic moment along negative x-axis, the magnetic force reaches a critical constant value when transverse diameter reaches 20 nm or thickness reaches 200 nm, at which magnetic force will not ever depend on sample dimension. Therefore, to quantify the size or resolute the image of ferromagnetic nano-domains, it is required for their dimension and geometry beyond a critical condition, which meanwhile depends on probe tip curvature and magnetic moment orientation. Especially, we could exploit the time-dependent magnetic force which is highly relying on magnetization intensity, as predicted by the variation profiles in Figure 4, to monitor magnetic nano-phase transformations on the magnetically unstable or catalytic surfaces [28]. For example, the dynamic process of phase transforming from Fe_3_O_4_ to Fe_2_O_3_ nanoparticles upon exposure to air could be characterized by the variation of magnetic force detected by MFM probe as a function of time, according to the results of MFM simulations, as illustrated in Figure 4.

**Figure 5:**
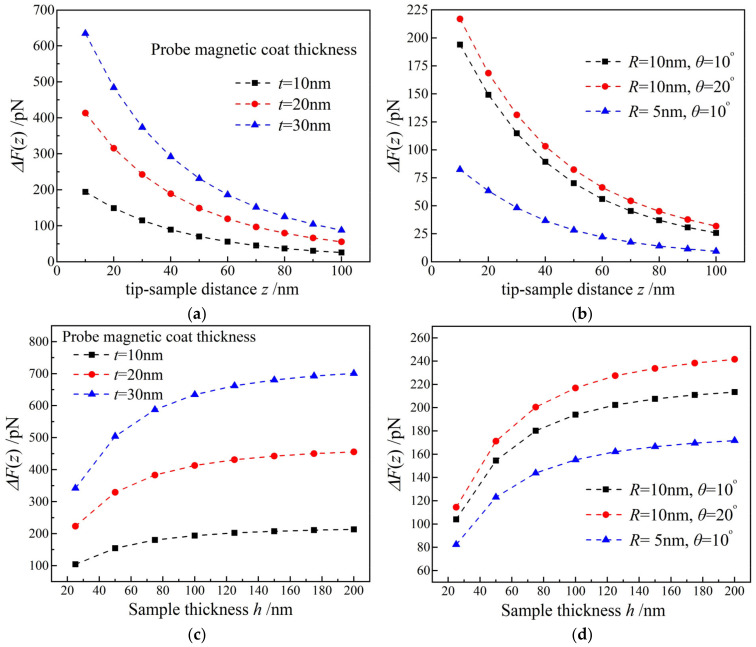
Magnetic forces varying with (**a**,**b**) distance between probe tip and sample; and (**c**,**d**) sample thickness.

The detected signal of MSM comes from magnetic force integration of magnetic coating on probe surface, which means that the accurate detection need to specify the thickness of probe magnetic coat, as shown in Figure 5a. The curvature of probe tip essentially determines MFM resolution of characterizing ferromagnetic nano-domains, as indicated in Figure 5b. The thicker magnetic coat on probe surface account for the magnetic force being more sensitive to tip-sample distance and sample thickness but will reduce tip curvature. Because the stray magnetic field decays away from sample surface in vertical direction (z-axis), only probe tip contributes to magnetic interaction between probe and sample, which accounts for the more and less dependence of magnetic force on tip curvature and truncated-cone angle respectively, as shown by Figure 5c,d. A finer tip of probe leads to a higher MFM resolution, but the excessively sharp probe tip is too vulnerable to be abraded in scanning process and cannot render a sufficient magnetic force, which will reduce measurement stability and restrain MFM imaging contrast, as illustrated in Figure 5b,d.

**Figure 6:**
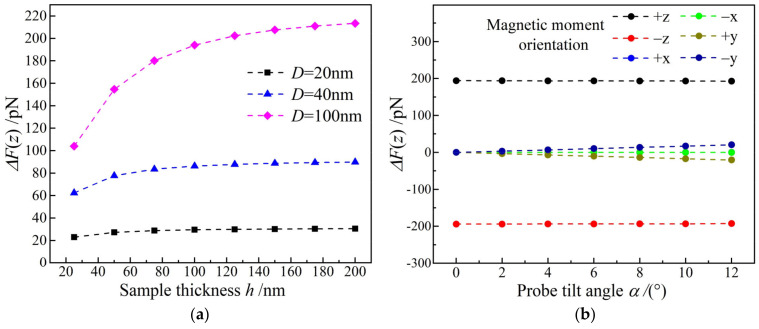
(**a**) Magnetic forces versus sample thicknesses for different sample diameters and a magnetic moment orientation along +z axis; (**b**) magnetic forces versus probe tilting angles along −y axis for samples (*h* = 100 nm, *D* = 100 nm) with different magnetic moment orientations.

Magnetic force is quite related to the shape and size of probe, the thickness of probe magnetic coat, the intensity and orientation of sample magnetic moment, and the distance and position of probe relative to the sample, and greatly dependent on sample thickness. As shown in Figure 6a, the critical sample thickness of the probed magnetic force reaching saturation relies significantly on sample transverse dimension, by which it can be concluded that critical thickness is approximately twice the transverse diameter of sample. Magnetic force signal also changes slightly when probe is tilted, as shown in Figure 6b. When sample magnetic moment (x-axis) is perpendicular to probe tilting plane (y-z), the magnetic force remains unchanged under probe inclination that is negligible for causing detection errors, as indicated by black and red curves in Figure 6b. Probe inclination leads to an increase in z-axis component of stray field that could be detected by probe, resulting in an appreciable linear increase of magnetic force with probe tilting angle when sample magnetic moment is along ±y axes.

### 3.2. Magnetic Field

**Figure 7:**
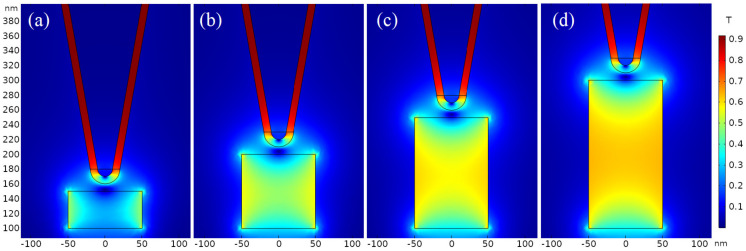
Magnetic flux density distribution detected by the probe of *H* = 1 μm, *θ* = 10°, *R* = 10 nm, *t* = 10 nm, *α* = 0° for ferromagnetic single-domains with a diameter of 100 nm and a thicknesses of (**a**) 50 nm; (**b**) 100 nm; (**c**) 150 nm; and (**d**) 200 nm.

Single-domain cylinder nanosamples of different thicknesses but with a magnetic moment along +z axis are taken as examples to analyze MFM magnetic forces of probe in stray magnetic fields of samples, as shown in Figure 7. Magnetic flux density near probe tip clearly depends on diameter/thickness ratio of single-domain nano-cylinders. For a nanosample with a smaller thickness, the magnetic flux density of the stray magnetic field on sample surface is almost identical to that of the whole sample as shown by Figure 7a, while for thicker samples as shown by Figure 7b–d, the magnetic flux primarily derived from the stray field on sample surface is concentrated on probe tip. When sample thickness is considerably smaller than diameter, the magnetic field in sample will almost be canceled by probe magnetic field, as manifested by the low magnetic flux density in Figure 7a. On the contrary, when sample thickness is significantly greater than the probe-sample spacing and reaches the critical thickness of approximately twice the sample diameter, as shown by Figure 7d, the magnetic flux inside sample is not affected by probe magnetism, implying that probe can only detect the stray magnetic field from sample surface, which accounts for a constant magnetic force without substantially varying with sample thickness.

### 3.3. Magnetic Force Probe Imaging

**Figure 8:**
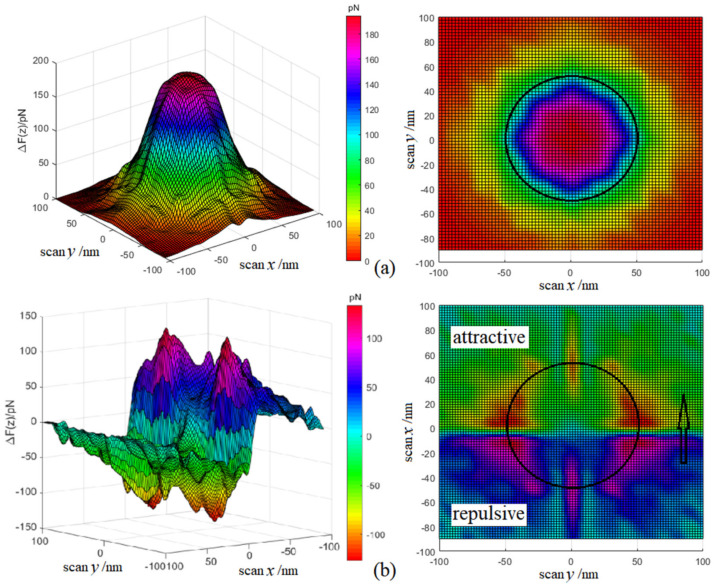
Magnetic force images of ferromagnetic nano-domains in 3D (left panels) and planar (right panels) maps for the tested samples with magnetic moments being (**a**) perpendicular and (**b**) parallel to sample surface.

Magnetic imaging for MFM is to date the second-order gradient mapping of magnetic forces, which is obtained by calculating the convolution of magnetic domains on probe tip and stray magnetic field on sample surface, which is inadequate for elucidating magnetic domain orientations and interpreting complex microscopic ferromagnetic structures. It is urgently necessary to characterize magnetic structures of ferromagnetic nano-domains, such as magnetic moment orientation, fine structure of magnetic domain, and stray magnetic field on sample surface. The shape of magnetic nano-domain is quite related to its magnetic moment orientation. Therefore, the magnetic moment orientation of a single-domain nanosample (cylinder *h*, *D* = 100 nm) is specified to be vertical (+z) and parallel (−x) with sample surface, respectively, and the scanning process is simulated by changing x and y coordinates of probe. When the magnetic moment of nanosample is in +z direction, the probed magnetic force reaches maximum at sample center and gradually decreases to zero at sample edge, as shown in Figure 8a, which can clearly characterize the size and shape of ferromagnetic nano-domains. When the magnetic moment of nanosample along negative x-axis is parallel to sample surface, a magnetic force morphology is imaged by probe scanning, which oscillates along magnetic moment orientation through sample center and approaches attractive and repulsive maxima, respectively, at two edges of magnetic nano-domain, as shown in Figure 8b. Thus, magnetic domain size can be estimated from the distance between two maximum positions, whilst magnetic moment orientation can be well determined by the morphological symmetry of magnetic force images, as illustrated by Figure 8b.

## 4. Conclusions

Magnetostatics 3D finite-element simulations of MFM characterizing single-domain ferromagnetic nanoparticles are performed, as implemented by AC/DC code of COMSOL Multiphysics 5.6, to systematically analyze magnetic forces between the magnetic-coated probe and the magnetic nano-domain by integrating Maxwell stress tensor on probe surface. For detecting magnetic properties of nanomaterials or the imaging characterizations of ferromagnetic nano-domains, it is required for sample thickness to be greater than a critical value, otherwise the detected magnetization and orientation of magnetic moment will be significantly interrupted by the limited longitudinal size of nanosamples. In particular, the probe detection of magnetic forces depends not only on the thickness and magnetization of permanent magnetic coat on probe surface, but also on tip geometry, probe tilt angle and sample thickness. Magnetic force direction alters greatly with sample magnetic moment orientation. When sample thickness is several times its transverse dimension, the measured magnetic force reaches the saturation state, which is no longer related to sample thickness. When sample magnetic moment is parallel or antiparallel to probe tilting direction, the probed magnetic force increases with the increase of probe inclination, but when perpendicular to probe tilting plane, the magnetic force signal is not interfered by probe inclination. Magnetic probe imaging can accurately characterize the sizes and magnetic moment orientations of ferromagnetic nano-domains under critical size requirements. Simulation analyses of microprobe magnetic forces in the present study render a technical strategy and a theoretical basis for developing magnetic probe microscopy to characterize magnetic structures of ferromagnetic nanomaterials.

## Data Availability

Theoretical methods and results are available from all authors.
